# Feminizing Adrenal Carcinoma Presenting with Heart Failure and Ventricular Tachycardia

**DOI:** 10.1155/2012/760134

**Published:** 2012-06-26

**Authors:** Anjana Harnoor, R. Lee West, Fiona J. Cook

**Affiliations:** Division of Endocrinology, Department of Internal Medicine and The Department of Pathology, Brody School of Medicine at East Carolina University, Greenville, NC 27834, USA

## Abstract

We present a case of feminizing adrenal carcinoma with severe elevation in serum estradiol and otherwise unexplained congestive heart failure with ventricular arrhythmia and review the literature on feminizing adrenal tumors and the potential relationship between estrogen and cardiac problems. A 54-year-old man presented with congestive heart failure and ventricular arrhythmia. Imaging revealed a large adrenal mass. Hormonal evaluation revealed a very high serum level of estradiol, elevated DHEA-sulfate and androstenedione, and lack of cortisol suppression on a low-dose overnight dexamethasone suppression test. The patient underwent a left adrenalectomy with subsequent normalization of serum estradiol. Surgical pathology examination established adrenocortical carcinoma MacFarlane stage II. Upon 15-month followup, the patient continued to have a normal serum estradiol level, his cardiac function was significantly improved, and he had no further episodes of ventricular arrhythmia. To the best of our knowledge, the serum estradiol level that was detected in our case is the highest that has been reported. Further, we hypothesize that the very high serum concentration of estradiol in our case may have played a role in his cardiac presentation with congestive heart failure and arrhythmia, particularly as these problems resolved with normalization of his serum estradiol level.

## 1. Introduction

Estradiol-secreting adrenal tumors are rare but are included in the differential diagnosis of gynecomastia. We present a case of a man with feminizing adrenocortical carcinoma associated with extremely high levels of estradiol. We review the literature on the potential effects of estradiol on cardiac function and cardiac arrhythmias. 

## 2. Case Presentation

A 54-year-old African American male with a 2-year history of hypertension, heart failure, and diabetes mellitus presented with progressive dyspnea and edema. He was evaluated for decompensated heart failure and had episodes of nonsustained ventricular tachycardia. An echocardiogram revealed dilated left-sided chambers and moderate global hypokinesis with ejection fraction of 35–40% (Table 1). He was treated and stabilized and transferred to our institution for consideration of internal defibrillator placement.

 Upon further questioning, the patient reported a 20 lb weight loss, decreased libido, erectile dysfunction, and breast development for the past 1-2 years.

Physical examination revealed stable vital signs (blood pressure 122/66, pulse 100 and regular) and normal weight (body mass index 23). At the time of our evaluation, he did not have rales on lung examination, S3 gallop, or peripheral edema. He had no Cushingoid features. Examination of the chest demonstrated gynecomastia and a hyperdynamic precordium. Examination of the abdomen revealed a firm mass in the left upper quadrant. Genitourinary examination was significant for soft 20 mL testes with no masses. 

Imaging studies showed an elevated left hemidiaphragm on chest radiograph and a large abdominal mass (12 × 11 × 17 cm) in the left upper quadrant on CT scan. This mass displaced the left kidney and was felt to arise from the left adrenal gland. Serum estradiol level was 3853 pg/mL, (normal range < 52 in males); this level was confirmed on repeat. Other pertinent hormonal evaluation included lack of cortisol suppression after low-dose dexamethasone, mildly elevated 24 hour urine free cortisol, elevated DHEA-S and androstenedione, and suppressed plasma ACTH (Table 2). 12 lead electrocardiogram showed normal sinus rhythm with a prolonged corrected QT interval of 477 msec and nonspecific anterior T wave abnormalities. 

**Figure 1:**
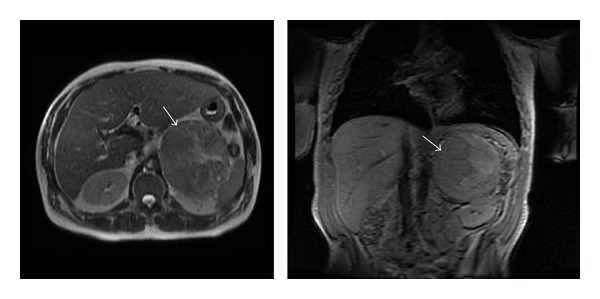
Magnetic resonance image of the abdomen demonstrating the left adrenal mass in transverse and coronal planes.

**Figure 2:**
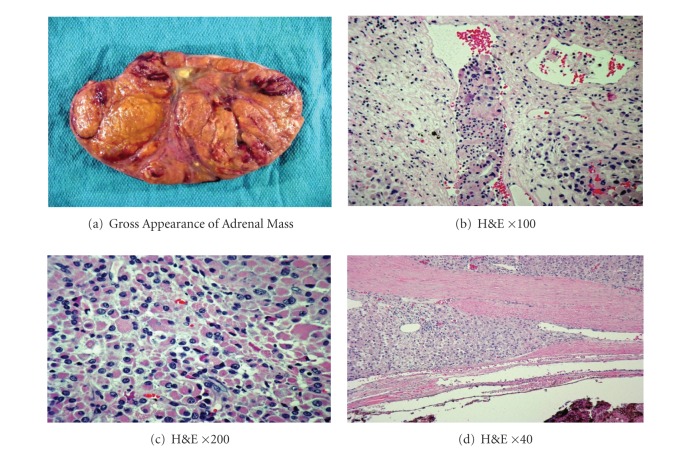
(a) Adrenocortical carcinoma weight 932 grams, (b) lymphovascular invasion, (c) oncocytic cells with frequent mitotic figures, and (d) capsular invasion.

Chest CT, abdominal MRI (Figure 1), and bone scan revealed no apparent metastases. Tumor resection and removal of the left adrenal gland, left kidney, spleen, and distal pancreas were performed. Pathology demonstrated a 932 gm adrenocortical carcinoma measuring 17 cm × 12 cm × 9 cm (Figure 2). Microscopic examination of the adrenal tumor revealed diffuse growth pattern, vascular invasion, necrosis, broad fibrous bands, and capsular invasion. Immunohistochemical stains showed the tumor cells to be positive for inhibin, MART-1, and negative for chromogranin, EMA, and pankeratin AE1 : 3, with adequate controls. Biopsy of ten regional lymph nodes was negative for malignancy. The pathologic stage was pT2, pN0, and pMx. The patient received stress dose steroids with a rapid taper to physiologic replacement. He did well postoperatively, and his estradiol level declined to <30 pg/mL. The patient was discharged on carvedilol, furosemide, metformin, glipizide, and NPH insulin with plans for further followup and treatment at another medical center. However, he was unable to afford to follow through with this. 

The patient returned to our endocrinology clinic 15 months following his surgery. He had noted improvement in gynecomastia. He denied palpitations, dyspnea, orthopnea, or chest pain and had not required any treatment for heart failure or arrhythmia in the interim. He continued on carvedilol, furosemide, glipizide, and NPH insulin. His weight had remained stable. Physical examination was remarkable for blood pressure 107/65, pulse 79 and regular, mild gynecomastia, normal cardiovascular and respiratory examination, and no peripheral edema. Serum estradiol level remained normal (Table 2). Unfortunately other adrenal hormone levels could not be retested due to financial constraints. On follow-up 12-lead electrocardiogram, corrected QT interval was normal at 396 msec. His diuretic was discontinued. He did not redevelop any signs or symptoms of heart failure. Echocardiogram done 2 months later revealed significant improvement in cardiac function with an ejection fraction of 60–65% and normal left ventricular dimensions, but persistent left atrial dilatation (Table 1).

## 3. Discussion

Adrenocortical carcinomas (ACC) are rare, with 1-2 cases per million patients [1]. Adrenal steroid hormone excess is evident in 40–60% of cases [1]. Cushing's syndrome is the most frequent presentation [1]. The incidence of feminizing ACC is much lower, being 1-2% of all ACC [2]. Feminizing ACC in males leads to gynecomastia, diminished libido and sexual potency, feminizing hair change, and testicular atrophy [2]. Our patient's serum estradiol level was extremely high at 3853 pg/mL, with normal for a male <52 pg/mL. The highest estradiol level previously reported in a male patient with adrenal carcinoma is 1065 pg/mL [2].

In assessing adrenocortical carcinoma, consideration is given to characteristics suggestive of poor prognosis. These include older age at presentation, tumor size > 6 cm, MacFarlane Stage III-IV, elevated level of estrogen, mixed excretion of other steroids, nuclear atypia and capsular or vascular invasion, and persistence or recurrence of elevated hormone levels [2]. Our patient's tumor was well over 6 cm, was MacFarlane stage II, and had autonomous secretion of cortisol and nuclear atypia and capsular and vascular invasion (Figure 2). Serum estradiol level was extremely high pre-op, but undetectable post-op.

Interestingly, our patient presented with congestive heart failure (CHF). It has been noted in the literature that males with CHF with low or high levels of estradiol demonstrate increased mortality when compared to the mean [3]. A case report from 1962 describes a case of CHF felt to be induced by high-dose estrogen in the form of stilbestrol, used at that time for treating duodenal ulcers. The signs and symptoms of CHF resolved with no other therapy than rest and withdrawal of estrogen [4]. In a study from 1953 using very high doses of stilbestrol to treat breast cancer, five patients who had no evidence of CHF prior to treatment died of CHF after treatment. The mechanism was felt to be estrogen-induced salt and water retention [5]. 

Zhan et al. studied the dose effect of estradiol replacement on mortality and cardiac remodeling and dysfunction post-myocardial infarction in ovariectomized mice. Estradiol tended to be cardioprotective at a low dose, but was cardiotoxic at supraphysiologic levels. The highest dose of estradiol increased plasma estrogen 8.5 fold [6]. This estradiol concentration does not approach the same magnitude found in our patient.

Our patient was transferred to our hospital for cardiac arrhythmia. Gender differences in cardiac electrophysiology have been reported for over a century [7]. Effects of sex hormones on cardiac electrophysiology have been studied in humans, animals, and cell models. In males, electrophysiologic differences become apparent during adolescence when the QT interval shortens. This has been correlated to testosterone levels. Estrogen has the opposite effect. QT intervals have been found to slightly increase with estrogen replacement therapy in postmenopausal women [8]. Inappropriate sinus tachycardia, AV nodal reentry tachycardia, sick sinus syndrome, idiopathic right ventricular tachycardia, and long QT arrhythmias are more common in women [7].

In a recent editorial review, Ciacco highlighted the relationship between torsades de pointes, sex hormones, and ventricular repolarization. He pointed out that women are 2-3 times more likely to experience episodes of torsades compared to men, risk of torsades increases with prolonged QT interval over 500 ms, and females have longer baseline QT intervals [9]. Elevated estrogen levels reduce potassium channel currents in a dose-dependent manner, causing action potential duration, and thus repolarization, to be delayed, which increases susceptibility to arrhythmia [8, 10]. Women appear to be more susceptible to drug-induced torsades [8]. A recent study done in model cells and tissues suggested high estradiol concentrations increased susceptibility to arrhythmias via QT prolongation [10]. Chen et al. found a higher incidence of torsades in dogs receiving cisapride (a drug known to increase QT interval) and estradiol than in those receiving cisapride alone. The change in QT with high dose estradiol alone was approximately 25 msec—this was with average plasma estradiol level 1615 pg/mL [11]. The change in our patient's QT interval between his pre- and post-op electrocardiogram suggests estrogen may have been affecting his cardiac repolarization. However, the effects of very high levels of estrogen on the heart are still poorly understood. 

The possible contribution of other adrenal hormones to our patient's cardiomyopathy cannot be ruled out. It is possible that cortisol excess, as demonstrated by an abnormal result on 1 mg dexamethasone suppression testing, as well as a suppressed plasma ACTH level, was a factor in his cardiac presentation; however, urine free cortisol was only moderately elevated at 56.7 mcg/24 hrs (normal 4–50.0). Cardiac involvement in Cushing's syndrome occurs primarily in the form of left ventricular hypertrophy and diastolic dysfunction [12]. There have been a few case reports of Cushing's patients with dilated cardiomyopathy, which was fully reversed after treatment [13]. The mechanism of this dilated cardiomyopathy has not been defined. The fact that our patient's serum aldosterone was low at <1 ng/dL (normal ≤ 28 upright) rules out aldosterone excess. The fact that his plasma renin was not suppressed suggests that it was unlikely that a different mineralocorticoid such as deoxycorticosterone was present in excess. Deoxycorticosterone was not measured in our patient.

The patient also had elevated levels of DHEA-S 726 mcg/dL (normal 25–240) and androstenedione 375 ng/dL (normal 50–220). DHEA-S level normalized after surgery. Studies have suggested that among men, low DHEA-S levels have been consistently associated with an increased risk of all-cause mortality and cardiovascular disease [14, 15]. We found no studies correlating high levels of DHEA-S and cardiac disease.

We acknowledge that diabetes and hypertension could have contributed to this patient's cardiomyopathy; however, these causes alone would not explain the significant improvement in his cardiac function after surgery for his adrenal tumor.

## 4. Conclusion

An important take home message of this case is that a middle-aged man with new onset gynecomastia should be evaluated with a hormonal panel to search for pathologic causes, including an estradiol level to screen for the rare case of a feminizing tumor.

In addition, this case highlights the possibility that the patient's uniquely high serum level of estradiol due to adrenal carcinoma may have contributed to arrhythmia by prolonging his QT interval, which normalized with normalization of serum estradiol level after surgery. It is also possible that the high concentration of estrogen contributed to his congestive heart failure due to dilated cardiomyopathy, which also resolved with normalization of serum estradiol. This unique clinical model appears to support previous observations on the cardiac effects of estrogen.

## Figures and Tables

**Table 1:** Echocardiography results.

	Preoperation	17-month postoperation
Left atrium dimension (cm) (normal 3.0–4.0 cm)	“moderately enlarged”	3.9
Left atrium vol/index (mL/m^2^) (normal 22 ± 6 mL/m^2^)	“moderately enlarged”	41
Left ventricle dimension (cm) (normal 4.2–5.9 cm)	7.1	5.1
Estimated ejection fraction (normal 50–80%)	35–40%	60–65%
Estimated pulmonary artery pressure (mm Hg) (normal 9–18 mm Hg)	35–40	23.7

**Table 2:** Hormonal evaluation.

	Pre-operation	Immediately postoperation	At 15-month followup	Reference Range
ACTH (pg/mL)	<5			7–50
AM cortisol (*μ*g/dL)	27.7			
Cortisol after overnight 1 mg dexamethasone suppression (*μ*g/dL)	23.6			<1.8
Androstenedione (ng/dL)	375			50–220
DHEA-S (*μ*g/dL)	726	39		25–240
Plasma metanephrine (pg/mL)	<25			≤57
Plasma normetanephrine (pg/mL)	<25			≤148
Total metanephrines (pg/mL)	<50			≤205
Aldosterone (ng/dL)	<1			≤28 (upright 8 am–10 am)
Plasma renin activity (ng/mL/hr)	2.8			0.65–5.0 (upright)
Estradiol (pg/mL)	3853	<30	25.2	<52 (males)
24 hr urine free cortisol (mcg/24 hrs)	56.7	Not measured	Not measured	4.0–50.0
24 hr urine volume (mL)	2080			
